# Resolvin E1 and Cytokines Environment in Skeletally Immature and Adult ACL Tears

**DOI:** 10.3389/fmed.2021.610866

**Published:** 2021-06-02

**Authors:** Marco Turati, Silvia Franchi, Giulio Leone, Massimiliano Piatti, Nicolò Zanchi, Marta Gandolla, Luca Rigamonti, Paola Sacerdote, Laura Rizzi, Alessandra Pedrocchi, Robert J. Omeljaniuk, Giovanni Zatti, Antonio Torsello, Marco Bigoni

**Affiliations:** ^1^Orthopedic Department, San Gerardo Hospital, Monza, Italy; ^2^School of Medicine and Surgery, University of Milano-Bicocca, Monza, Italy; ^3^Transalpine Center of Pediatric Sports Medicine and Surgery, University of Milano-Bicocca - Hospital Couple Enfant, Monza, Italy; ^4^Transalpine Center of Pediatric Sports Medicine and Surgery, University of Milano-Bicocca - Hospital Couple Enfant, Grenoble, France; ^5^Department of Pediatric Orthopedic Surgery, Hopital Couple Enfants, Grenoble Alpes University, Grenoble, France; ^6^Department of Pharmacological and Biomolecular Sciences, University of Milan, Milan, Italy; ^7^NearLab, Department of Electronics, Information, and Bioengineering, Politecnico di Milano, Milan, Italy; ^8^Department of Orthopedic Surgery, Mayo Clinic, Rochester, MN, United States; ^9^Department of Biology, Lakehead University, Thunder Bay, ON, Canada

**Keywords:** cytokines, anterior cruciate ligament, resolvin, adolescent, knee, synovial

## Abstract

The intra-articular synovial fluid environment in skeletally immature patients following an ACL tear is complex and remains undefined. Levels of inflammatory and anti-inflammatory cytokines change significantly in response to trauma and collectively define the inflammatory environment. Of these factors the resolvins, with their inherent anti-inflammatory, reparative, and analgesic properties, have become prominent. This study examined the levels of resolvins and other cytokines after ACL tears in skeletally immature and adult patients in order to determine if skeletal maturity affects the inflammatory pattern. Skeletally immature and adult patients with an anterior cruciate ligament injury and meniscal tears were prospectively enrolled over a 5-month period. Synovial fluid samples were obtained before surgery quantifying Resolvin E1, IL-1β, TNF-α, and IL-10 by ELISA. Comparisons between skeletally immature patients and adults, the influence of meniscal tear, growth plate maturity and time from trauma were analyzed. Skeletally immature patients had significantly greater levels of Resolvin E1 and IL-10 compared with adults with an isolated anterior cruciate ligament lesion. Among the injured skeletally immature patients Resolvin E1 levels were greater in the open growth plate group compared with those with closing growth plates. Moreover, levels of Resolvin E1 and IL-10 appeared to decrease with time. Our results suggest that skeletally immature patients have a stronger activation of the Resolvin pattern compared to adult patients and that synovial fluid Resolvins could play an antinflammatory role in the knee after anterior cruciate ligament lesion and that its activity may be synergistic with that of IL-10.

## Introduction

Anterior cruciate ligament (ACL) rupture is a frequent injury in young and active subjects who practice sports such as soccer, rugby, American football, basketball, and skiing (1). In the last several decades the frequency of ACL injury in skeletally immature patients has increased due to (i) younger participation in sports and (ii) increased numbers of young athletes (2, 3).

The management of pediatric ACL injuries is currently a matter of vigorous debate (4). The International Olympic Committee (IOC) consensus statement about pediatric ACL treatment stresses the importance of high-quality rehabilitation programs with or without ACL reconstruction for pediatric patients with isolated ACL tears (5).

Meniscal tears, often associated with ACL injuries, typically involve damage to the lateral meniscus (6). In pediatric cases involving a repairable meniscus, ACL surgical reconstruction is recommended. Although, different ACL reconstruction techniques are available for the pediatric population, long-term follow-up results are still unknown. In the case of injured adults, it is known that injured ACL, and/or untreated meniscal tears involve a high risk of developing post-traumatic osteoarthritis (OA) (7); however, despite surgical reconstruction, there is still a higher risk of developing OA than in the healthy knee (8). To illustrate, over 40% of patients presented radiographic evidence of post-traumatic OA 7 to 11 years following ACL rupture (9); in comparison, in patients with ACL rupture associated with meniscal tear the occurrence of OA is almost 70% (7, 10).

The relationship between age, ACL injuries, and OA incidence was recently investigated and no differences were found in knee OA at 15 years after adolescent ACL reconstruction compared with the adult cohort based on radiographic image analysis (11).

In adults, the pro-inflammatory cytokines arising post-lesion predispose the subsequent development of OA; by contrast, data in pediatric patients are still lacking (12–14).

Recently, it has been suggested that the shift from an inflammatory- to a non-inflammatory state may be mediated by a coordinated and regulated program. During the inflammatory phase several specific mediators are synthesized which may modulate or even terminate the inflammatory mechanism and thereby promote tissue repair. In addition to anti-inflammatory cytokines, resolvins (a family of lipid mediators) are emerging as one main class of injury resolution molecules. Resolvin E is derived from eicosapentaenoic acid (EPA), whereas, Resolvin D is derived from docosahexaenoic acid (DHA). Resolvin E is endowed with powerful anti-inflammatory, reparative, and analgesic actions in various diseases exhibiting inflammatory pathogenesis (15). Failed activation of resolution mechanisms may be responsible for chronic inflammation and consequent tissue injury. In this study we investigated synovial fluid concentration changes of pro-inflammatory cytokines (IL-1β, TNF-α), an anti-inflammatory cytokine (IL-10) and Resolvin E1 (RvE1) in skeletally immature knees with ACL tears, comparing them with those measured in adult knees with ACL tears.

The purpose of this study was to examine whether resolvins play a role in inflammation after ACL tears, with or without meniscal tears, and if there are specific inflammatory patterns in skeletally immature and adult patients. At present there are no published data about levels of resolvins in the synovial fluid of individuals with isolated ACL rupture or associated with meniscal tears. We hypothesize that resolvins may play important roles countering inflammation and promoting tissue healing after a traumatic knee injury.

## Materials and Methods

### Study Design and Population

Patients with an ACL rupture which required surgical treatment were recruited into this single-center study between December 2017 and April 2018 and assorted into an (i) adolescent group and an (ii) adult group. The adolescent group consisted of patients between 10 and 17 years old bearing a traumatic ACL tear (16) whereas, the adult group consisted of patients ≥24 years of age with a similar history of knee trauma and ACL tear.

Exclusion criteria included (i) any other associated ligamentous injury requiring surgical management, (ii) chondral and osteochondral lesions, (iii) bone fractures, (iv) degenerative meniscal tears, prior knee surgery, previous meniscal, or ACL injury to the same knee, (v) intraarticular or extra-articular chronic inflammatory disease, (vi) autoimmune disease, or neoplasia, (vii) use of immunomodulatory drugs or aspirin, intraarticular injection of corticosteroids and other drugs, and (viii) radiologic and arthroscopic signs of OA.

Synovial fluid was taken from the affected knee of each patient at the time of arthroscopic surgery. A diagnosis was made by orthopedic examination, then confirmed with MRI and arthroscopic procedure. Concomitant injury to the meniscus was determined arthroscopically at the time of ACL reconstruction.

Growth plate maturity was evaluated on the basis of anterior-posterior knee radiographs. Distal femoral growth plates were classified using the Walls Classification system into three types: (i) open, (ii) closing, and (iii) closed. Closing growth plates were those showing both areas of open and closed physis (17, 18).

Samples were assorted into groups according to the time elapsed between injury and fluid collection including (i) acute (A, between 0 and 48 h after injury), (ii) early subacute (ESA, between 3 and 15 days after injury), (iii) late sub-acute (LSA, between 15 days and 3 months after injury), and (iv) chronic (C, more than 3 months after injury) (19).

All patients and parents provided written informed consent. The experimental protocol was approved by the local Ethical Committee and conforms to the principles outlined in the WMA Declaration of Helsinki.

### Synovial Fluid Collection and Storage

Synovial fluid sampling was performed after sterile preparation with chlorhexidine using the medial or superolateral access with a 20-gauge needle. Synovial fluids were transferred into 15 ml sterile tubes, stored on ice, and transferred to the laboratory, where they were centrifuged at 3,000 g to eliminate cells and other debris. Supernatants were frozen and stored at −80°C until assayed.

### Biomarker Analysis

Levels of RvE1 and IL-1β, TNF-α and IL-10 in synovial fluid were quantified using commercially available ELISA (Enzyme-Linked Immuno-Sorbent Assay, or immuno-absorbent test linked to an enzyme) kits, following the protocol provided by the manufacturers (IL-1 β, IL-10, and TNF-α were from R&D Systems, Minneapolis, MN, USA; RvE1 from MyBioSource, San Diego, USA). All samples were run in duplicate. Levels are expressed as pg/ml.

### Statistical Methods

Data analysis was run with non-parametric statistics being data non-normally distributed (as confirmed by Shapiro-Wilk test), and being this approach not affected by extreme values and more robust to outliers. Comparisons between skeletally immature patients and adults, and between isolated ACL rupture and ACL rupture associated with meniscal tears were made using the two-way analysis of variance (ANOVA), that examines the influence of two different categorical independent variables (type of lesion and type of patient) on one continuous dependent variable (biochemical marker). The Mack-Skillings statistical test is a non-parametric two-way ANOVA used for unbalanced incomplete block designs, when the number of observations in each treatment/block pair is one or greater. Effect of time was investigated through the Mack-Skillings statistical test with two different categorical independent variables (timing and type of patient) on one continuous dependent variable (biochemical marker). When significant, *post-hoc* analysis was run in different groups with Mann-Whitney test applying Bonferroni correction. The influence of the Wall classification for skeletally immature patients was analyzed by the Kruskall Wallis test (3 groups). When significant, *post-hoc* analysis was run in different groups with the Mann-Whitney test applying Bonferroni correction. Finally, the correlations between molecular concentrations were made using the Spearman correlation. For all tests, statistical significance was assumed at *p* < 0.05.

## Results

### Subjects

Characteristics of patients are reported in Table 1. Of the 59 patients recruited, the adolescent group included 29 patients (9 females and 20 males) with a mean age of 15.62 years (SD = 1.44; range: 12–17 years) at the time of synovial fluid collection. The adult group included 30 patients (8 females and 22 males) with a mean age of 34.8 years (SD = 6.85; range: 24–47 years).

**Table 1:** Patient's characteristics by adolescent and adult groups.

	**Adolescent**	**Adult**
	**(*n* = 29)**	**(*n* = 30)**
Age at surgery, years	15.6 ± 1.4	34.8 ± 6.8
Female patients, *n* (%)	9 (31%)	8 (27%)
Time from trauma to synovial fluid collection (days)	49 ± 59.9	103 ± 66.8
Isolated ACL tear, *n* (%)	14 (48.3%)	13 (43.4%)
Concomitant Meniscal tear, *n* (%)	15 (51.7%)	17 (56.6%)
Physis status, *n* (%)		
Open	4 (13.8%)	/
Closing	10 (34.5%)	/
Closed	15 (51.7%)	/

At the time of surgery, a concomitant meniscal tear was confirmed in 15 adolescent (51.7%) and 17 adult (56.6%) patients.

The mean time between injury and synovial fluid sampling for the population was 76 days (SD = 114.9; range: 1–493 days). For the adolescent group, the mean interval was 49 days (SD = 59.9; range: 1–267 days), while for the adult group the mean interval was 103 days (SD = 66.8; range: 1–493 days). For the adolescent group, we observed four patients with open growth plates (two with associated meniscal tear), 10 with closing growth plates (six with concomitant meniscal tear) and 15 patients with closed growth plates (seven of them with concomitant meniscal tear).

### Cytokines and Resolvin Levels in Adolescent and Adult ACL Tears

Levels of RvE1, IL-1β, IL-10, and TNF-α were measured by ELISA in knee synovial fluid samples of all adolescent and adult patients (Table 2).

**Table 2:** Cytokine levels in synovial fluids from adolescent and adult ACL Tear with or without a concomitant meniscal tear.

	**Adult Isolated ACL**		**Adult ACL tear with a**		**Adolescent Isolated**		**Adolescent ACL tear with a concomitant**	
	**tear (*****N*** **=** **13)**		**concomitant Meniscal tear (*****N*** **=** **17)**		**ACL tear (*****N*** **=** **14)**		**Meniscal tear (*****N*** **=** **15)**	
	**Mean**	**SD**	**Mean**	**SD**	**mean**	**SD**	**Mean**	**SD**
RvE1	79,326	68,518	63,650	52,694	471,863	291,627	163,765	97,130
IL-1β	1,146	0,211	1,425	0,648	1,643	0,741	1,092	0,218
IL-10	4,643	1,582	3,906	0,805	13,142	8,172	6,497	2,384
TNF-α	22,865	12,524	15,692	8,366	25,922	11,484	15,811	4,043

*Cytokine concentrations (pg/ml). SD, standard deviation*.

**Figure 1:**
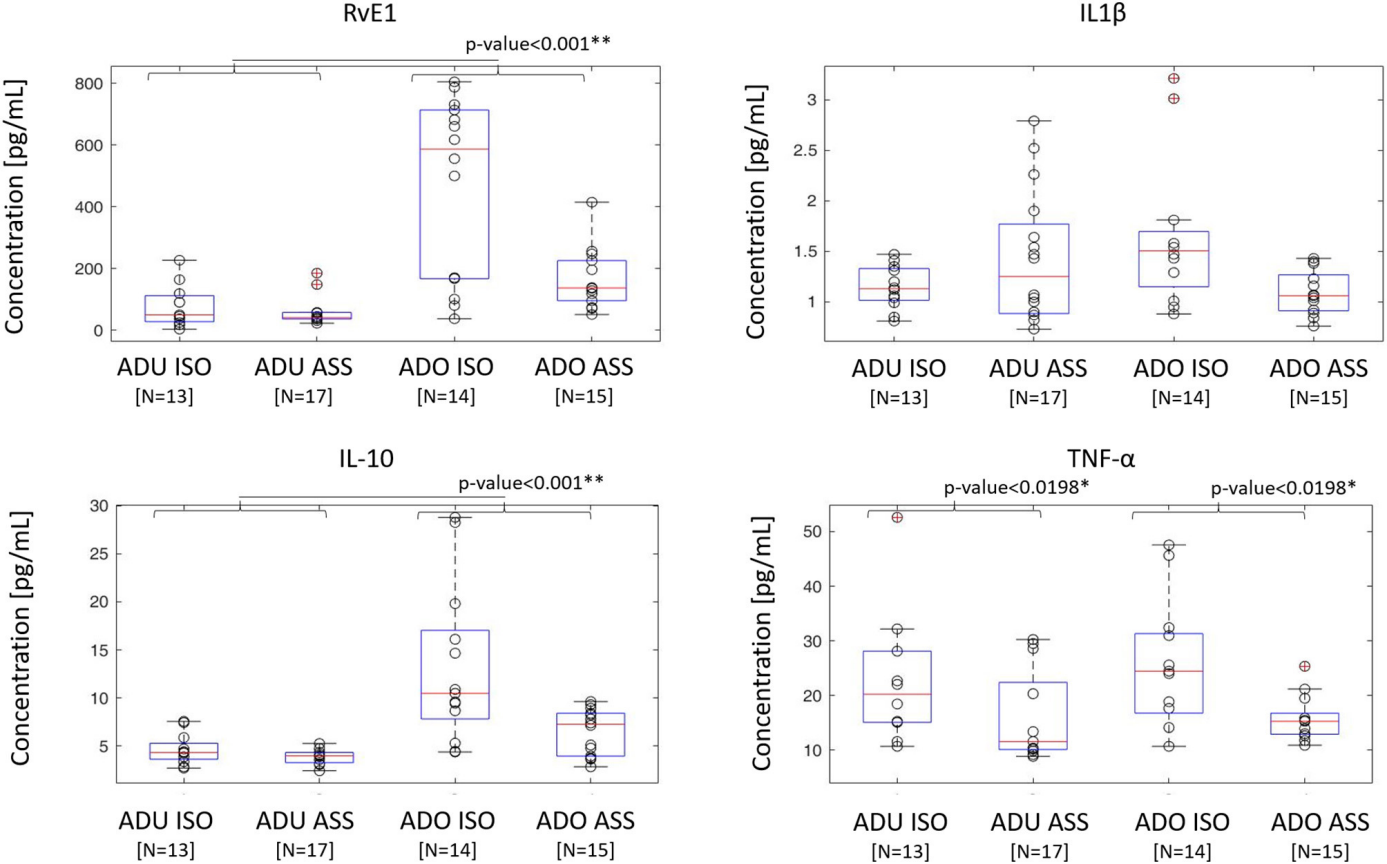
Cytokine and resolvin concentrations in adult and adolescent groups with isolated or meniscal associated ACL. Cytokine and RvE1 concentrations measured in the adolescent group (*n* = 29) and adult group (*n* = 30) are represented in the box plot. ISO, isolated ACL rupture; ASS, ACL rupture associated with meniscal tears. Gray dot represents concentrations of each single patient. In each box plot, the box is built within the third (upper bound) and first (lower bound) quartiles (i.e., *Q*_3_, *Q*_1_); the middle line represents the median. Whiskers represent data maximum (upper whisker) and minimum (lower whisker). Defined as data points below *Q*1–1.5 × (*Q*3–*Q*1) or above *Q*3 + 1.5 × (*Q*3–*Q*1). *Indicates statistical significance (*p* < 0.05). **p < 0.001.

The two-way analysis of variance (ANOVA) examined the influence of age (adolescent and adult cohort) and concomitant meniscal tear on one continuous dependent variable (synovial fluid biochemical level). We applied the Mack-Skillings statistical test, that is a non-parametric two-way ANOVA used for unbalanced incomplete block designs, when the number of observations in each treatment/block pair is one or greater (20). In adolescents with an ACL tear, synovial fluid levels of IL-10 and RvE1 were significantly greater (*p* < 0.05) than those in the adults with an ACL tear (Figure 1). Moreover, in both the adult and adolescent groups, TNF-α levels were significantly greater in isolated ACL tear samples (*p* < 0.05) compared with those in an ACL tear with a concomitant meniscal tear.

### Effects of Growth Plate Maturation on Cytokine and Resolvin Levels in Adolescent With ACL Tear Population

**Figure 2:**
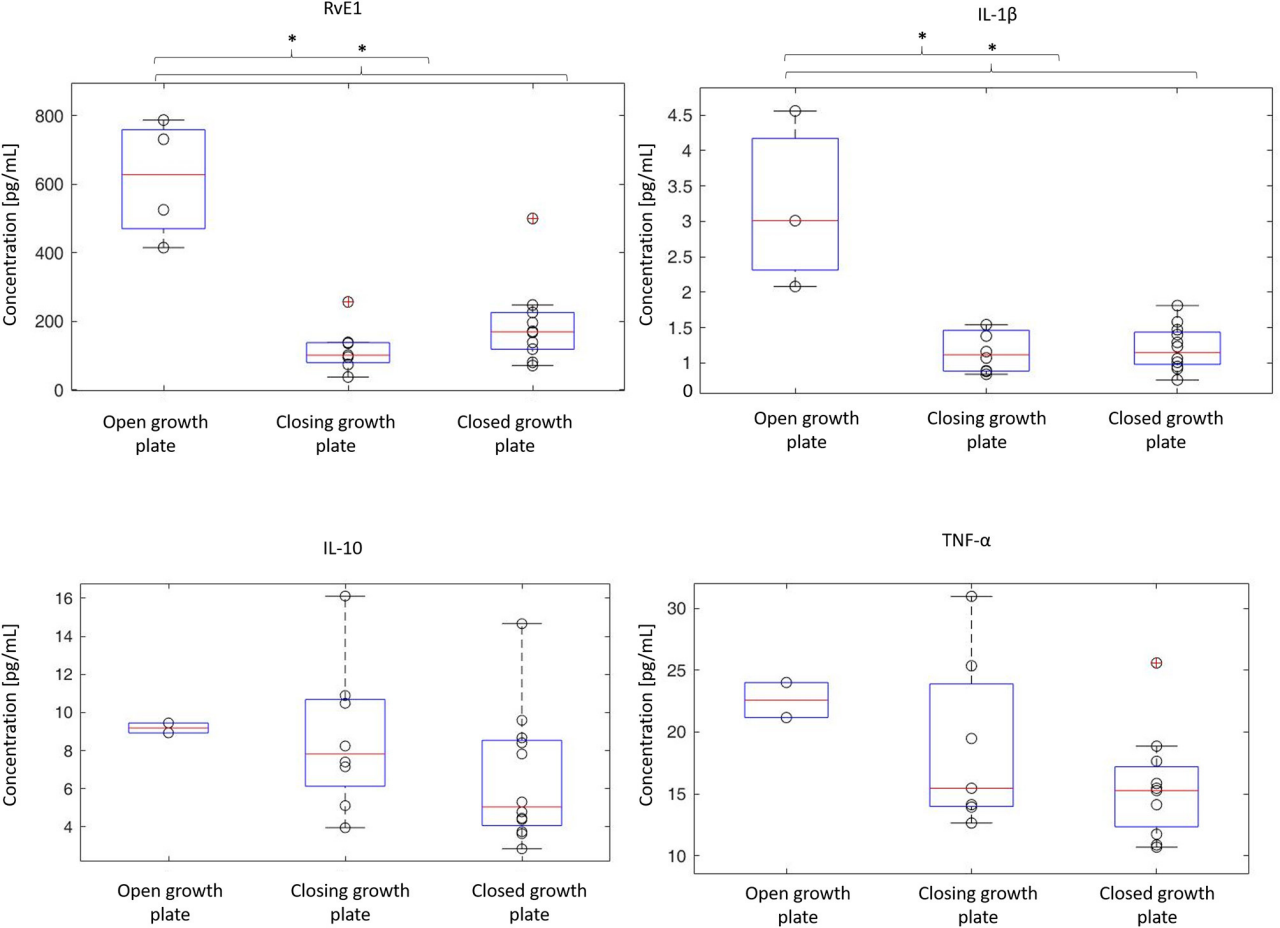
Modifications of cytokine and resolvin levels in relation to growth plate maturity. Growth plate maturity was assessed on anterior-posterior knee radiographs. Femoral growth plates were classified into three types (4 patients with open, 10 with closing and 15 with closed growth plates) (17, 18). Cytokine and RvE1 concentrations measured in the adolescent cohort with ACL tear (*n* = 29) are represented in the box plots. Gray dot represents cytokine concentrations of each single patient. In each box plot, the box is built within the third (upper bound), and first (lower bound) quartiles (i.e., *Q*3, *Q*1); the middle red line represents the median. Whiskers represent data maximum (upper whisker) and minimum (lower whisker). Defined as data points below *Q*1–1.5 × (*Q*3–*Q*1) or above *Q*3 + 1.5 × (*Q*3–*Q*1). *Indicates statistical significance (*p* < 0.05).

Among the adolescent groups, RvE1 and IL-1β concentrations were significantly greater in open growth plate subjects compared to those with a closing growth plate (*p* < 0.001) and closed growth plate (*p* < 0.001) (Figure 2). The levels of IL-10 and TNF-α did not significantly vary among adolescent patients according to growth plate status.

### Effects of Time Between Injury and Synovial Fluid Sampling on Cytokine and Resolvin E1 Concentrations in Adolescents and Adults With ACL Tears

The effect of time elapsed from ACL injury to sampling was examined through the Mack-Skillings statistical test with two different categorical independent variables (i.e., timing from ACL injury, type of patient) on one continuous dependent variable (i.e., biochemical marker), which both resulted to be significant.

**Figure 3:**
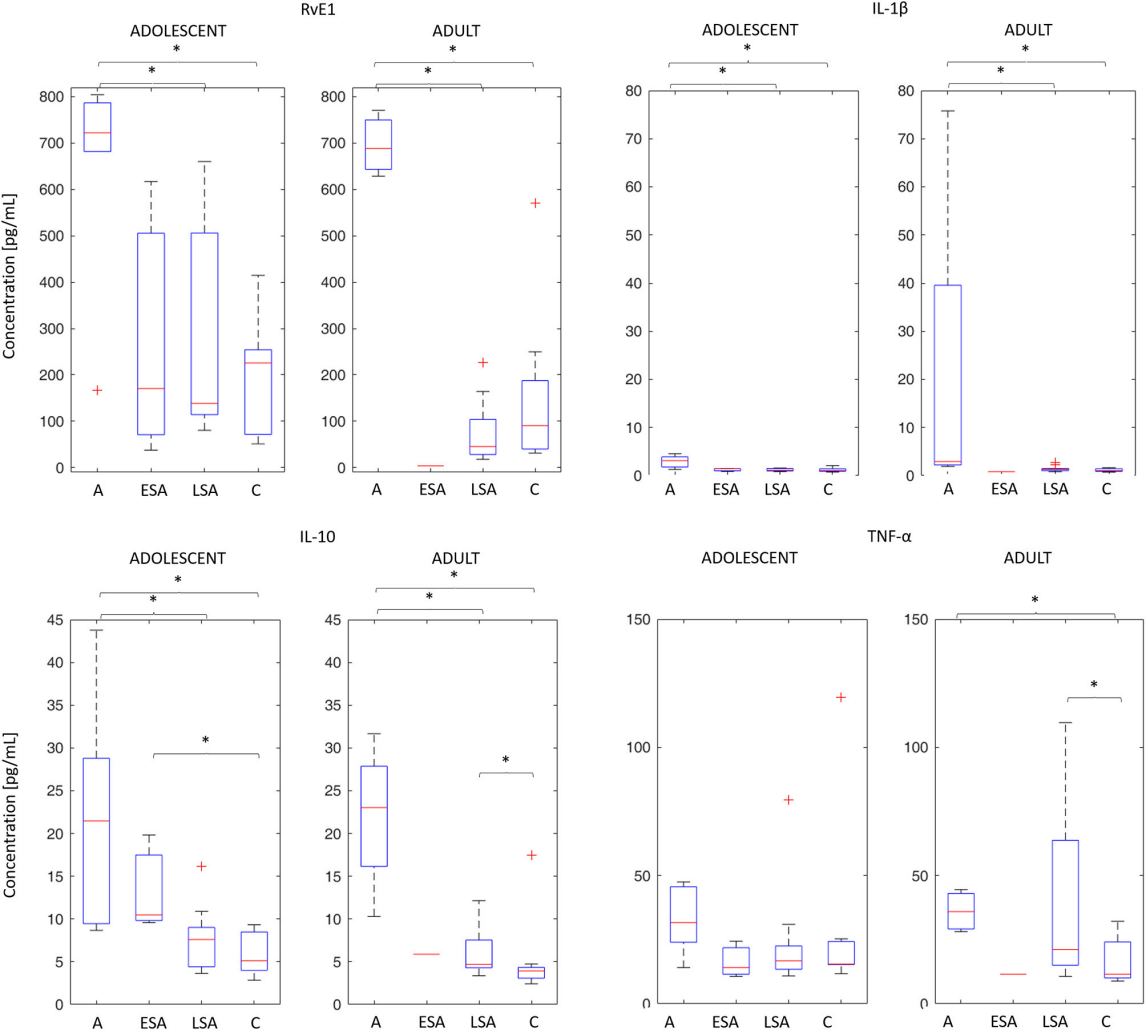
Time-dependent modifications of cytokine and resolving levels in adolescent and adult cohorts. The median concentration (horizontal line), box plot, and 95% CI of the studied biomarkers have been plotted for the following time points: acute (A), early sub-acute (ESA), late sub-acute (LSA), and chronic (C), as defined in Materials and Methods Section. *Indicates statistical significance (*p* < 0.05).

In the *post-hoc* analysis, interestingly RvE1, IL-1β, and IL-10 levels significantly decreased over time (*p*-values < 0.05) in both the adolescent and adult groups (Figure 3). Notably, concentrations of TNF-α decreased significantly in the adult group alone (*p* < 0.05).

### Correlations Between Cytokines and RvE1 in Adolescent and Adult Knees With an ACL Tear

Correlations among RvE1 and cytokine levels in synovial fluid were investigated independently of concomitant meniscal tear and growth plate maturity in both groups. In the adolescent group the levels of IL-1β were positively correlated with those of RvE1 (*p* ≤ 0.0001, *r* = 0.66), IL-10 (*p* ≤ 0.0001, *r* = 0.66), and TNF-α (*p* = 0.0084, *r* = 0.49). Moreover, the levels of IL-10 were also positively correlated with those of RvE1 (*p* = 0.0310, *r* = 0.41) and TNF-α (*p* = 0.0366, *r* = 0.40). In the adult group levels of IL-1β were positively correlated with those of TNF-α (*p* = 0.009, *r* = 0.49) and IL-10 (*p* = 0.0153, *r* = 0.46) in the absence of any correlation with any other species (Table 3).

**Table 3:** Correlations between cytokines and Resolvin E1 in the synovial fluid of adolescent and adult ACL tears.

	**RvE1 (pg/ml)**	**IL-1 (pg/ml)**	**IL-10 (pg/ml)**	**TNFα (pg/ml)**
**Rho values**				
RvE1 (pg/ml)	–	0.66	0.41	0.36
IL-1 (pg/ml)	0.22	–	0.66	0.49
IL-10 (pg/ml)	0.13	0.46	–	0.40
TNFα (pg/ml)	−0.04	0.49	0.33	–
***p*****-values**				
RvE1 (pg/ml)	1.0000	0.0001^*^	0.0310^*^	0.0609
IL-1 (pg/ml)	0.2732	1.0000	0.0001^*^	0.0084^*^
IL-10 (pg/ml)	0.5248	0.0153^*^	1.0000	0.0366^*^
TNFα (pg/ml)	0.8595	0.0090^*^	0.0950	1.0000

*Correlations between cytokines and Resolvin E1 (RvE1) in the synovial fluid of adolescent (29-white squares) and adult (30 - gray squares) ACL tears*.

* *Indicates statistical significance (p < 0.05). Correlations among cytokines were investigated using the non-parametric Spearman rank correlation coefficient test*.

## Discussion

An acute inflammatory response to an injury is meant to be protective and, ideally, self-limiting (21). Among the varied biochemical inflammatory responses to injury, there is a heightened focus on examining the anti-inflammatory properties and roles of resolvins (22). Children and adults differ in their immune system inflammatory responses to tissue injury (23) which predisposes children to faster and more effective healing than adults. Accordingly, this study examined and compared changes in concentrations of resolvin-E1 (RvE1) in addition to those of varied cytokines in the synovial fluids of children and adults consequent to ACL injury.

In cases of an isolated ACL injury, skeletally immature patients had significantly greater levels of RvE1 and IL-10 (*p* < 0.001) compared with adults. To the best of our knowledge, this is the first such documented observation. RvE1 plays an important role in inflammation regulation, polymorphonuclear neutrophils action (24) and even in inflammatory pain attenuation (25). Appropriate generation of this lipid mediator in the early to intermediate inflammatory process appears to be a prerequisite for a successful resolution of inflammation and tissue protection and may be a critical step in successful intra-articular repair. To illustrate, Dakin et al. observed that *in vitro* co-incubation of shoulder tendon cells (from patients with shoulder tendon tears) with RvE1 up-regulated pro-resolving inflammation patterns (26). Unfortunately, the specific roles and mechanisms of RvE1 in ACL and meniscal repair remain undefined.

Bigoni et al. recently reported the synovial fluid concentrations of IL-10 from 17 adolescent patients with ACL tears who had undergone ACL reconstruction. They concluded that the IL-10 concentrations in these adolescents were greater than those found in adults in comparable studies (18). Data from the present study supports that conclusion. These data are consistent with the phenomenon of superior ligament healing potential in adolescents compared with that of adults. IL-10 action is protective against joint damage with its anti-inflammatory properties (27). Furthermore, IL-10 inhibits activation of pro-inflammatory mediators including TNF-α, IL-1, IL-6, IL-8, IL-12 from monocytes/macrophages (28–30).

Findings from multiple studies suggest that growth potential and age may influence concentrations of specific cytokines (18, 31). For example, although, concentrations of IL-1β are greater in patients with open growth plates, there is apparently no correlation of IL-10 concentration with growth plate status (18). As well, Bigoni et al. (18) reported that concentrations of IL-8 and TNF-α levels are notably greater in patients with open femoral physis. The results of this study confirm and extend those observations; specifically, synovial fluid concentrations of RvE1, and IL-1β were significantly greater in patients with open growth plates compared with patients with closing growth plates (*p* = 0.006 for RvE1 and *p* = 0.012 for IL-1β) as well as those with closed growth plates (*p* = 0.004 for both RvE1 and IL-1β). By contrast, however, synovial fluid concentrations of TNF-α appear unrelated to growth plate status based on our data from a study involving a larger cohort of skeletally immature patients (19). Consequently, the extent to which physis development influences the intra-articular biochemical environment is becoming an important emerging concern mandating appropriate clinical and preclinical investigation.

Synovial fluid levels of RvE1 and IL10 progressively decreased over time post-injury in both adults and adolescents with an ACL tear. It is now well-established that the levels of anti-inflammatory IL-10 are elevated following trauma as well as surgical repair. However, these levels of IL-10 rapidly decrease thereafter leaving active inflammatory species present which potentiate an increased risk of articular damage in adults (13). Synovial fluid concentrations of RvE1 also appear to vary temporally with those of IL-10 in the skeletally immature patients in this study.

Regrettably, the effects and the timing of preservative knee surgery, including (i) meniscal repair during ACL reconstruction, (ii) proximal ACL reinsertion and (iii) bridge-enhanced suture repair on the absolute concentrations and temporal variations of these species remains to be investigated (32, 33).

This study, furthermore, presents various correlations of synovial fluid concentrations of these species in both adolescent and adults who have experienced ACL injury. In children levels of RvE1 were positively correlated with those of IL-1β (*p* ≤ 0.0001), as well as for IL-10 (*p* = 0.03) (Table 3). These correlations, not observed in the adult group, may support the concept that the posttraumatic inflammatory response differs considerably in its nature between children and adults.

Several factors contributing to the healing potential of the traumatized ACL have been examined. Murray et al. (34) found that the ACL remnant has healing potential after rupture, but that this process can be disturbed by synovial tissue lining over the injured surface. As well, biomechanical properties of the remnant have been reported; to illustrate, Georgulis et al. found mechanoreceptors in an ACL remnant 3 years after injury (35). Furthermore, Murray et al. (36) studied the structural properties of a bio-enhanced ACL repair in a pig model and found that those properties were not inferior compared to a reconstructed ACL. Muneta et al. (37), considered evidence regarding the utility of ACL remnant preservation during ACL reconstruction; although, several studies reported healing and mechanical properties of the remnant, there is, as yet, insufficient evidence to confirm or refute the benefit of remnant-preserving surgery. In the absence of conclusive evidence, there is still a suggestion that ACL reparative surgery may be useful in specific circumstances (e.g., precise nature of injury and patient's age).

ACL repair in children is still a matter of considerable debate with few studies available. Sherman et al. (38) reported 50 cases of ACL repair in both children and adults, with good results; however, age was not actively considered as a factor involved in repair success or failure. By comparison, we reported a 5-case series of proximal ACL injuries in children treated with a surgical ACL repair, with a 100% success rate (39). This kind of series suggests that the healing potential in children is a strong factor to consider in pre-operative surgical planning and repair has to be considered in cases of proximal lesions in young patients (40). By comparison, Gagliardi et al. reported a graft failure rate 10 times higher in skeletally immature patients that underwent ACL repair compared to ACL reconstruction. However, Patient Reported Outcome Measures of ACL repair without re-rupture were comparable to ACL reconstructed patients. In this context the real role of the biochemical environment associated with that ACL repair failure rate was unknown and not investigated (41). Although, more studies are needed to determine conclusively the best treatment options for certain lesions in certain age groups, ACL repair surgery must be strongly considered in young patients.

The resolution process during inflammation is complex, and many pro-resolving mediators, both lipids and proteins, are likely involved in addition to RvE1. For example, the D-series Resolvins have been shown to play a role in joint diseases such as arthrosis and may also be a relevant factor in the ACL micro-environment (42).

The most important strength of this study is its originality, since it describes for the first time in literature Resolvin levels in adolescents with an isolated ACL tear or an ACL tear associated with a meniscal lesion and the impact of time from lesion on this mediator. Nonetheless we are aware of some limitations of the study. First, a small number of patients enrolled due the strict inclusion/exclusion criteria as a specific diagnosis of traumatic ACL tear in a selected group of patient. The small sample size restricts the power of our statistical analyses and was a consequence of a lower than expected number of subjects enrolled during the recruitment period. However, the methodology selected for the analysis in this study was based on non-parametric tests that usually require smaller samples in order to be effective. In particular, the non-parametric analysis of variance (Mack-Skillings statistical test) is a generalization of the Friedman test when there are missing data (43). If we derive the achieved statistical power obtained using a two-way ANOVA with a total sample size of 59, a large desired effect size (0.4), and alpha equal to 0.05, we obtain a statistical power equals to 0.77, which is very close to the usual threshold set as 0.8. This means that it is less probable that we can discern an actual effect, but it also points toward the fact that if we detect a difference, it is likely to be biologically significant.

Despite the heterogeneity in skeletal maturation existing between males and females during adolescence, we included in the adolescent group patients between 10 and 17 years old without sex distinction. Considering the difficulties associated with recruitment, this choice allowed us to obtain a more substantial number of patients. Moreover, the adolescent and adult groups presented some other differences; in particular, the mean time between knee injury/sprain and synovial fluid sampling was shorter in the adolescent group than in the adult group. However, the RevE1 levels remains higher in adolescent patients than in adult patients when synovial fluid were obtained at the same time post lesion, suggesting that age rather than time is the relevant variable. A comparable trial, with greater numbers of patients and standard synovial fluid collection timing should nonetheless be undertaken in the future in order to improve our understanding of differences in cytokines and resolvins between adolescents and adults. Another limitation of this study may be associated with the gender imbalances within both adult (27% females) and adolescent (31% females) groups. We did not previously find any statistically significant differences between males and females in their synovial fluid IL1, IL10, and TNF-α concentrations (18); consequently, we consider that gender imbalance does not substantially undermine the statistical analyses of our data or conclusions arising therefrom in this study. Moreover, female representation within the adolescent- and adult-group were comparable. Finally, we regret that we do not present resolvin and cytokine data from healthy subjects. First of all, arthrocentesis is difficult to perform in non-swollen knees, to extract a meaningful amount of fluid is necessary to inject an amount of sterile saline solution before aspiration sampling with the consequent requirement of data adjustment to correct for dilution (44); then, it is also an exceptionally invasive technique which renders it ethically dubious for a healthy subject.

Notwithstanding these limitations, this study provides the first evidence that the synovial fluid of adolescent patients has significantly greater levels of the anti-inflammatory species RvE1 and IL-10 compared with those of adults with an isolated ACL lesion. RvE1 levels were greater in adolescents with open growth plates compared with those having closing, or closed, growth plates. Moreover, levels of RvE1 and IL-10 appear to decrease with time following injury. This finding implicates the involvement of RvE1 and IL-10 in the more robust healing potential observed in adolescents.

## Data Availability Statement

The raw data supporting the conclusions of this article will be made available by the authors, without undue reservation.

## Ethics Statement

The studies involving human participants were reviewed and approved by San Gerardo Hospital Ethic Committee. Written informed consent to participate in this study was provided by the participants' legal guardian/next of kin.

## Author Contributions

MB, MT, GL, LRig, AP, RO, MP, AT, and SF: design of the study. MB, MT, MP, GL, LRiz, NZ, PS, LRiz, and SF: performed the experiments. MG, AP, MT, GL, SF, MB, RO, AT, LRiz, MP, GZ, and NZ: analyzed the results. MT, SF, MB, GL, LRiz, AT, MG, NZ, MP, LRiz, and PS: drafting of the manuscript. MT, PS, GL, GZ, AT, RO, MB, LRig, MG, NZ, AP, and SF: manuscript final revision. All authors approved the final version of manuscript for submission.

## Conflict of Interest

The authors declare that the research was conducted in the absence of any commercial or financial relationships that could be construed as a potential conflict of interest.

## Abbreviations

ACL: anterior cruciate ligament
OA: osteoarthrosis
RvE1: Resolvin E1
IL-1β: interleukin 1β
TNF-α: tumor necrosis factor alpha
IL-10: interleukin 10
EPA: eicosapentaenoic acid
DHA: docosahexaenoic acid
MRI: magnetic resonance imaging.
